# Ambassadors in Dermatology and Venereology: An interview with Professor Fabio Ayala

**DOI:** 10.1111/jdv.20589

**Published:** 2025-03-25

**Authors:** Fabio Ayala

**Affiliations:** ^1^ University of Naples Federico II Naples Italy

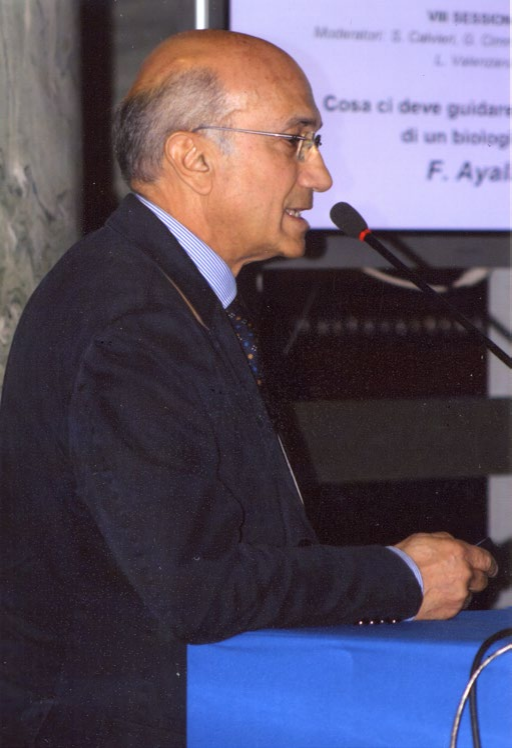

## CURRICULUM VITAE

Year of birth: 1947

## ACADEMIC EDUCATION

1971 Degree in Medicine and Surgery, University of Naples – Italy – Summa cum laude
1973 Specialist in Dermatology and Venereology, University of Naples – Italy – Summa cum laude
1972–1973 Fellow, University of Naples Federico II—Italy

## PROFESSIONAL CAREER

1974–1976 Clinical Instructor, University of Naples Federico II—Italy
1977–1982 Assistant Clinical Professor of Dermatology, University of Naples Federico II—Italy
1983–1990 Associate Professor of Dermatology, University of Naples Federico II—Italy
1990 (Nov)–1994 (Oct) Full Professor of Dermatology, University of Reggio Calabria—Italy
1994–2017 Full Professor of Dermatology, University of Naples Federico II—Italy
1994–1997 Member of the Board of the Italian National Institute of Health Council
1998–2001 Director, Resident School in Dermatology and Venereology
2002–2005 Director, Resident School in Plastic and Reconstructive Surgery
2005–2017 Head of the Dermatology Unit, University of Naples Federico II—Italy
2019 Professor Emeritus of Dermatology
2007–2017 Coordinator of the Dermatology Doctoral Programs, Doctoral School in Clinical and Experimental Medicine
2009–2022 Coordinator of the European Academy of Dermatology and Venereology (EADV) Contact Dermatitis Task Force (together with Prof. Ana Giménez‐Arnau)

## SOCIETIES AND MEMBERSHIP

- Italian Society of Dermatology and Venereology—SIDeMaST (formerly SIDEV) (1993–1999: Secretary, Board of Directors; 2007–2009 and 2012–2015: Treasurer)
- Italian Society of Allergological, Professional and Environmental Dermatology—SIDAPA (1999–2004: Board of Directors; 2005–2008: President)
- European Society of Contact Dermatitis—ESCD (1994–1998: Italian representative in the ESCD Council)
- European Academy of Dermatology and Venereology (EADV)
- European Society of Dermatological Research (ESDR)
- European Dermatology Forum (EDF)

## WHAT BROUGHT YOU TO DERMATOLOGY?

I was essentially born into the world of Dermatology, as both my grandfather and father were dermatologists. Growing up in Naples, I lived and breathed Dermatology from an early age. Our house was large and included the office where they ran their private practice, alongside their public work. My father, after serving as a university assistant, was the Head of the Dermatology Unit at Salerno Hospital for over 25 years. Perhaps for these reasons, my own son decided to follow in their footsteps and became a dermatologist 25 years ago.

## WHO WERE YOUR MOST IMPORTANT TEACHERS?

I had two main teachers: Pietro Santoianni and my father, Libero.

In 1973, when the Second Medical School was founded in Naples, Prof. Pietro Santoianni was appointed Head of the Dermatology Clinic, and I was named his first Assistant. He was an excellent researcher, and he served as Director until 2004, when I succeeded him as Head of the Department. Pietro Santoianni was fascinated by the new dermatological equipment that began appearing on the market since the 1970s. Our Department acquired the first Italian liquid nitrogen cryotherapy apparatus and the first Helio‐neon laser. Although excellent results on skin ulcers had been reported in the literature, we demonstrated the ineffectiveness of this kind of laser light. I presented these findings at a Gordon Research Conference in Chicago. I also recall that the first phototherapy equipment was purchased and used in our Department for both research and clinical applications starting in the 1970s.

As for my father, Libero, he was not only an exceptional teacher of dermatological practice but also an extraordinary life mentor. I began working in the Dermatology Unit in Salerno shortly before graduating, learning clinical and dermatological semiology at the patient's bedside. At that time in Italy, as in France, the importance of dermatological disease classification according to primary morphology prevailed, underlining the fundamental importance of basic lesions in achieving a correct diagnosis.

## WHICH SOCIETIES HAVE YOU BEEN ACTIVE IN, AND WHICH IS CLOSEST TO YOUR HEART?

The society I hold most dear is the Italian Society of Allergological, Occupational and Environmental Dermatology (SIDAPA). I served as a board member from 1999 to 2004 and later as President from 2005 to 2008. In this area of Dermatology, I also represented Italy on the Council of the European Society of Contact Dermatitis (ESCD) from 1994 to 1998.

Another society close to my heart is the Italian Society of Dermatology Medical, Surgical, Aesthetic and Sexually Transmitted Diseases (SIDeMaST). I was a board member and secretary from 1993 to 1999, and later served as treasurer from 2007 to 2009 and again from 2012 to 2015. This society represents the historical evolution of the Società Italiana di Dermatologia e Sifilografia (SIDES), one of the oldest European dermatological societies, founded in 1885.

In addition, I have been a member of other societies, including the European Academy of Dermatology and Venereology (EADV), the European Society of Dermatological Research (ESDR) and the European Dermatology Forum (EDF).

Please list your five best publications

- David Pesqué, Olivier Aerts, Mojca Bizjak, Margarida Gonçalo, Aleksandra Dugonik, Dagmar Simon, Suzana Ljubojević‐Hadzavdić, Laura Malinauskiene, Mark Wilkinson, Magdalena Czarnecka‐Operacz, Beata Krecisz, Swen M. John, Anna Balato, Fabio Ayala, Thomas Rustemeyer, Ana M. Giménez‐Arnau. Differential diagnosis of contact dermatitis: A practical‐approach review by the EADV Task Force on contact dermatitis. *J Eur Acad Dermatol Venereol* (2024) 38:1704–1722.
- Marcella Nunziato, Anna Balato, Anna Ruocco, Valeria D'Argenio, Roberta Di Caprio, Nicola Balato, Fabio Ayala, Francesco Salvatore. A Familial Novel Putative‐Pathogenic Mutation Identified in Plaque‐Psoriasis by a Multigene Panel Analysis. *Int J Mol Sci* (2023) 24:4743–4750.
- A. Balato, E. Scala, F. Ayala, A. Bauer, M.‐N. Crépy, M. Gonçalo, J. Duus Johansen, S.M. John, T. Rustemeyer, N. Wagner, M. Wilkinson, A. Giménez‐Arnau. Patch test informed consent form: position statement by European Academy of Dermatology and Venereology Task Force on Contact Dermatitis. *J Eur Acad Dermatol Venereol* (2021) 35:1957–1962.
- Annunziata Raimondo, Serena Lembo, Roberta Di Caprio, Giovanna Donnarumma, Giuseppe Monfrecola, Nicola Balato, Fabio Ayala, Anna Balato. Psoriatic cutaneous inflammation promotes human monocyte differentiation into active osteoclasts, facilitating bone damage. *Eur J Immunol* (2017) 47:1062–1074.
- Anna Balato, Martina Mattii, Giuseppina Caiazzo, Annunziata Raimondo, Cataldo Patruno, Nicola Balato, Fabio Ayala, Serena Lembo. IL‐36γ Is Involved in Psoriasis and Allergic Contact Dermatitis. *J Invest Dermatol* (2016) 136:1520–1523.

## WHAT MOTIVATED YOU TO BECOME ACTIVE IN SOCIETY POLITICS?

The main reason was the need to highlight the diverse aspects of Dermatology and its relationship with many internal diseases. This effort aimed to counter the perception of Dermatology as merely a specialty focused on creams and lotions. A few decades ago, this was a common view among other specialists, who considered Dermatology as the “Cinderella” of medical fields. Today, however, Dermatology has become one of the most sought‐after specialties in Italy, ranking second in demand among medical graduates.

## WHAT ADVICE WAS MOST HELPFUL FOR YOUR CAREER?

Perhaps the most valuable advice was to choose a degree thesis on a topic in biological chemistry, the results of which were published in *Biochimica et Biophysica Acta* under the title “Studies on the activation of lysine‐2,3‐aminomutase by S‐adenosyl‐L‐methionine” in 1972.

As a result, I attended the Biochemistry Institute of the University of Naples from 1968 to 1971. I was fortunate enough to work in an institute where many young researchers later secured prestigious positions in universities and research centres both in Italy and abroad. Working firsthand with centrifuges, chromatography equipment and mass spectrometers at a time when such instruments were seldom used in dermatological research, I was in a position to set up subsequent research with a wealth of technical knowledge that was not common to young dermatologists at the time. Later, this facilitated me to carry out research in the field of porphyrias and photodermatoses, using also simple equipment developed according to the indications of our Institute.

## WHAT WAS THE GREATEST ACHIEVEMENT IN YOUR PROFESSIONAL LIFE?

My career developed progressively, beginning with a research scholarship. Shortly after becoming a confirmed researcher, I realized that by studying and working hard, I could advance my career and eventually lead a dermatology department.

This was my main aspiration and, once I achieved this goal, I consistently worked, along with my collaborators, to help medical students appreciate the fascinating nature of skin diseases and the importance of interdisciplinary collaboration. This has always been the best approach for the diagnosis and treatment of skin diseases, especially those linked to internal medicine.

## WHAT DO YOU LIKE BEST IN YOUR PROFESSION?

In addition to my teaching and clinical research activities, I have always maintained close contact with patients through my private dermatology practice. My research activity has always had practical implications and applications for the treatment of skin diseases.

Even after retiring a few years ago, I have continued to collaborate with the clinic I led for about 15 years, both to stay updated on developments and to assist with patients requiring specialized clinical or laboratory investigations.

What has always fascinated me most is solving problems of research and diagnosis in difficult clinical cases. Now that I can focus mainly on private practice, I prefer patients with difficult dermatoses, perhaps because, as a teenager, I was an avid fan of crime fiction. Let me explain: solving a difficult clinical case feels much like unravelling the plot of a crime novel or film, where the “killer” (the hidden disease) is discovered by putting together the “clues” (clinical signs, symptoms and analysis results).

## WHAT WAS THE MOST DIFFICULT FIGHT/TASK IN DERMATOLOGY POLITICS FOR YOU?

The main challenge I faced as Head of the Dermatology Department was ensuring that there were enough staff to support the work, especially in the later years. The secretarial staff, nurses and porters were retiring without being replaced. At the same time, the demand for hospitalizations and examinations in various, sometimes newly established, sections was increasing, while staff numbers continued to decline. Unfortunately, the hospital administration was unable to recruit and allocate the necessary personnel. As a result, the workload for all staff, including doctors, progressively grew.

## WHAT WAS THE GREATEST DISAPPOINTMENT IN YOUR PROFESSIONAL LIFE?

I must say I consider myself very fortunate in my professional life, likely thanks to my calm approach, prudence, and courage in dealing with different situations. I have always been a realist, rather than a dreamer, which has helped me avoid significant disappointments. By cautiously anticipating the potential outcomes of my actions, I have managed to navigate challenges without encountering major setbacks.

## CAN YOU SHARE ANY FUNNY EPISODES FROM YOUR PROFESSIONAL LIFE?

In the past, my own androgenetic alopecia created some discomfort in patients who came to me with similar concerns about hair loss. I was a young dermatologist and the patients, even young ones, were distressed by their problem. When they entered my office and saw that I had the same issue, they were sometimes sceptical: how could a doctor who hadn't been able to treat his own alopecia possibly help them? On top of that, as is often the case in Italy, patients were sometimes accompanied by their mothers, who seemed even more perplexed. It required a certain level of skill to regain their trust and explain in detail what they could expect while highlighting the importance of collaboration between patient and dermatologist.

## WHO HELPED YOU MOST IN YOUR STRUGGLES FOR DERMATOLOGY?

I don't think I have ever had to face any major battles in my profession or my life. This is because the family I was born into, as well as the family that came later, have always been very supportive and never hindered me in my pursuits. On the other hand, as already mentioned, my career has been relatively smooth, with varying paces of progress but no significant obstacles. Of course, I was fortunate to have the close bond that existed between my father, my mother, my two sisters and me. A perfect example of the old saying “unity is strength”.

## APART FROM DERMATOLOGY, WHAT IS YOUR MAJOR INTEREST?

Classical music has always been a big part of my life. I played classical guitar for several years as a child and teenager. Even when I was in elementary school I often listened to classical music while studying. I think it's the only type of music that complements study and work, without distracting from these activities. And even today, I work and study with classical music playing in the background.

In addition, Naples is a coastal city, allowing me a very close relationship with the sea. Over the years, I have gradually moved from small motorboats to sailboats. Eight years ago, I decided to sell my last 38‐feet sailboat. Since then, I have preferred to charter catamarans or sloops around the world. In the past, with our sailboats, we would spend weekends visiting nearby islands and take short holidays to explore more distant ones. Sailing is a fascinating sport even though it does not allow you to cover long distances in a short time, but it does offer the opportunity for diving or simply go snorkelling.

## WHAT IS YOUR FAVOURITE WRITER, COMPOSER, AND PAINTER?

- Writer: I don't choose a book based on its author, but rather on its subject. I prefer rather short books, contemporary novels, with an analysis of the characters' personalities. I also prefer a good movie or a theatre play over a book and never choose science fiction topics.
- Composer(s): Georg Friedrich Händel, Johann Sebastian Bach.
- Painter(s): Pablo Picasso for his extraordinary imagination, Henri Matisse as the emotional expression of the Fauves, Mark Rothko for his formidable combination of colours.

## WHERE DO YOU SEE THE GREATEST PROBLEMS FOR OUR SPECIALTY IN THE NEXT 10 YEARS?

I think it is really hard to predict the next 10 years, so I'd focus on the next five.

First, new equipment that has recently come onto the market is very expensive, and at least in our country, few institutions can afford it, which limits its use.

In addition, in public and often private universities and hospitals, waiting lists are growing, which hinders the timely diagnosis and treatment of skin diseases—conditions that actually require prompt intervention.

I have the impression that dermatologists today have less interest in certain areas that are our expertise and that must remain in our field. For example, I believe that Allergy and Professional Dermatology are going through a difficult time, both due to the declining interest of young specialists and the well‐known European legislative difficulties for the production and use of haptens for patch tests.

## WHAT ADVICE WOULD YOU LIKE TO GIVE TO YOUNG COLLEAGUES?

Today, in Italy, senior dermatologists have observed that in recent years residents are different from the past: they are quick and technologically skilled, but they often fail to dedicate the necessary time to find solutions to problems. This can sometimes be the case even when they are caring for patients. I think this reflects how the new generations approach life today. They are often distracted by chats and messages, their minds are driven to search for quick answers to questions that suddenly come to mind while reading, talking or studying. This is often a different kind of focus compared to what we were used to. This is obviously one of the differences that exist and will always exist between different generations and it's likely that we must adapt to these changes. However, I believe that excessive speed and the tendency to be distracted are not compatible with finding optimal solutions to patients' problems.

I have one more piece of advice: choose a specific area of interest within Dermatology and make it a point to stay updated in that field without losing sight of the broader scope of Dermatology as a whole. I think this is the only way forward: united, ensuring that our specialty stands firm against the danger of losing knowledge and leadership in the management of skin diseases.

## CONFLICT OF INTEREST STATEMENT

None declared.

